# MHA Herbarium: Collections of mosses from Yana-Indigirka Region, Yakutia, Russia

**DOI:** 10.3897/BDJ.10.e77341

**Published:** 2022-01-21

**Authors:** Michael S. Ignatov, Elena A. Ignatova, Elena I. Ivanova, Vera G. Isakova, Oleg V. Ivanov, Alexey P. Seregin

**Affiliations:** 1 Lomonosov Moscow State University, Moscow, Russia Lomonosov Moscow State University Moscow Russia; 2 Tsitsin Main Botanical Garden, Moscow, Russia Tsitsin Main Botanical Garden Moscow Russia; 3 Institute for Biological Problems of Cryolithozone of Siberian Division of Russian Academy of Sciences, Yakutsk, Russia Institute for Biological Problems of Cryolithozone of Siberian Division of Russian Academy of Sciences Yakutsk Russia; 4 Lebedev Institue of Physics of Russian Academy of Sciences, Moscow, Russia Lebedev Institue of Physics of Russian Academy of Sciences Moscow Russia

**Keywords:** occurrence, specimen, NE Asia, Verkhoyansk Mountain Range, Bryophyta, new records

## Abstract

**Background:**

The Skvortsov Herbarium of the Tsitsin Main Botanical Garden, Russian Academy of Sciences (MHA) in the 1945-1980s dealt with vascular plants and only scattered occasional collections of bryophytes and lichens were accumulated there without special arrangement. Since the late 1980s, the bryophyte studies in the MHA Herbarium became permanent and several projects were started since then, including the currently conducted “Moss Flora of Russia”. There are many white spots on the map of bryophyte exploration of Russia, but one of the most conspicuous was Yakutia, the largest administrative unit of Russia, covering 3,081,000 km^2^. Yana-Indigirka Region, originally defined as a floristic region, includes Verkhoyansky Range and some smaller adjacent mountain areas. It is the largest amongst the bryofloristic regions in Russia, but exploration of its territory, which is difficult to access, remains far from complete.

**New information:**

Several expeditions of the Institute for Biological Problems of Cryolithozone, Siberian Branch of Russian Academy of Sciences, and the Main Botanical Garden, Russian Academy of Sciences in 2000-2018 yielded in many bryophyte specimens, partly published in a number of papers. This dataset comprehensively represents the diversity of mosses of the Region. It includes 7,738 records of moss specimens preserved in the MHA Herbarium.

## Introduction

Yana-Indigirka Region of Yakutia is an area defined by Karavaev (1958) for its mainly mountain region eastwards of Lena River and its largest right side tributary, the Aldan River. The original definition was only slightly modified by Kuznetsova (2005), but otherwise it remains in current use for biodiversity studies of Yakutia (Ivanova et al. 2005) and also it is used as one of subdivisions of Russian territory for the Moss Flora of Russia (Ignatov 2017, Ignatov 2018, Ignatov 2020) and its database (Ivanov et al. 2017). Covering over one million km^2^, this floristic region is the largest amongst regions in both Yakutia and the whole of Russia.

Accumulation of data on the moss flora of Yana-Indigirka Region of Yakutia started relatively late compared to its other parts, because early exploration of Yakutia was conducted either along the Arctic Ocean coast accessible by ships (Arnell 1913) or in Aldan Region in the southern Yakutia by expeditions of Kuzeneva and Prokhorov (Brotherus et al. 1916). Exploration of vegetation in many areas of Yakutia in the 1950-1980s brought scattered collections from many localities, but mostly of widespread moss species. In the 1980-1990s, bryofloristic studies became more numerous; their scope is overviewed by Ivanova et al. (2005). Collections accumulated in Herbaria of the Institute for Biological Problems of Cryolithozone, Siberian Branch of Russian Academy of Sciences (SASY) and Komarov Botanical Institute, Russian Academy of Sciences (LE). Since 2000, collаboration between Institute for Biological Problems of Cryolithozone SB RAS and the Main Botanical Garden, Russian Academy of Sciences started.

## General description

### Purpose

The general aim of the whole study was to fill a considerable gap in knowledge of moss diversity in the "cold pole" of the Northern Hemisphere. After preliminary studies, it turned out that many moss species in the huge permafrost area of Asia remained undescribed, being erroneously named by existing identification manuals of Europe, Japan and North America. The latter flora were based on local material and results in taxonomy, though carefully done, were inapplicable to the permafrost area of Yakutia. As usual, taxonomic studies require abundant material, from regions with as much diversity as possible. Therefore, a number of expeditions were further conducted in different areas of Yakutia in 2000-2018.

## Project description

### Title

MHA Herbarium: Collections of mosses from Yana-Indigirka Region, Yakutia, Russia

### Study area description

**Figure 1.**
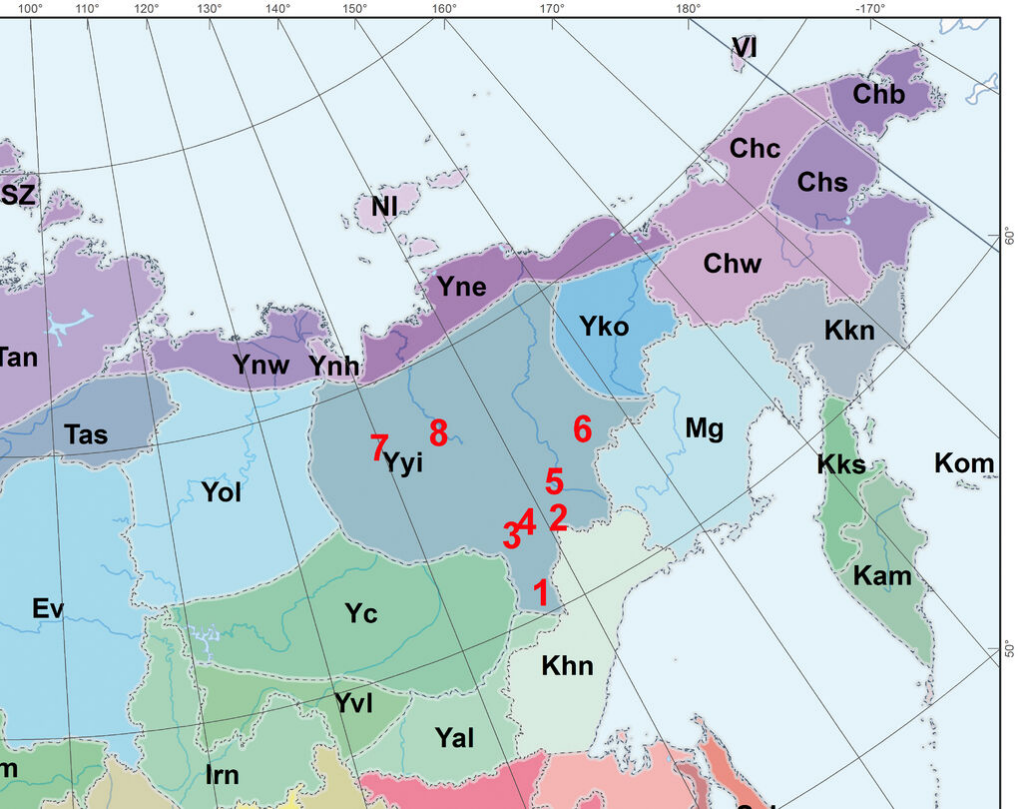
Areas studied for moss floras in the Yana-Indigirka floristic region (Yyi). Each area includes a number of localities along 100–300 km field trips, with more exact collecting sites provided in respective publications for each of them: **1** Yudoma-Maya Plateau and Tarbagannakh Mountain (Ignatov et al. 2001); **2** Mus-Khaya Peak surroundings (Ignatova et al. 2011); **3** Sette-Daban Range (Ignatova et al. 2018); **4** Suntar-Khayata Nature Reserve (Ivanova et al. 2016); **5** Ust-Nera area (Ivanova et al. 2018); **6** Ulakhan-Chistai Range (Ignatova et al. 2020); **7** Orulgan Range around upper course of the Tumara River (Ignatov et al. 2014); **8** Yana-Adycha Plateau (Isakova 2010).

**Figure 2.**
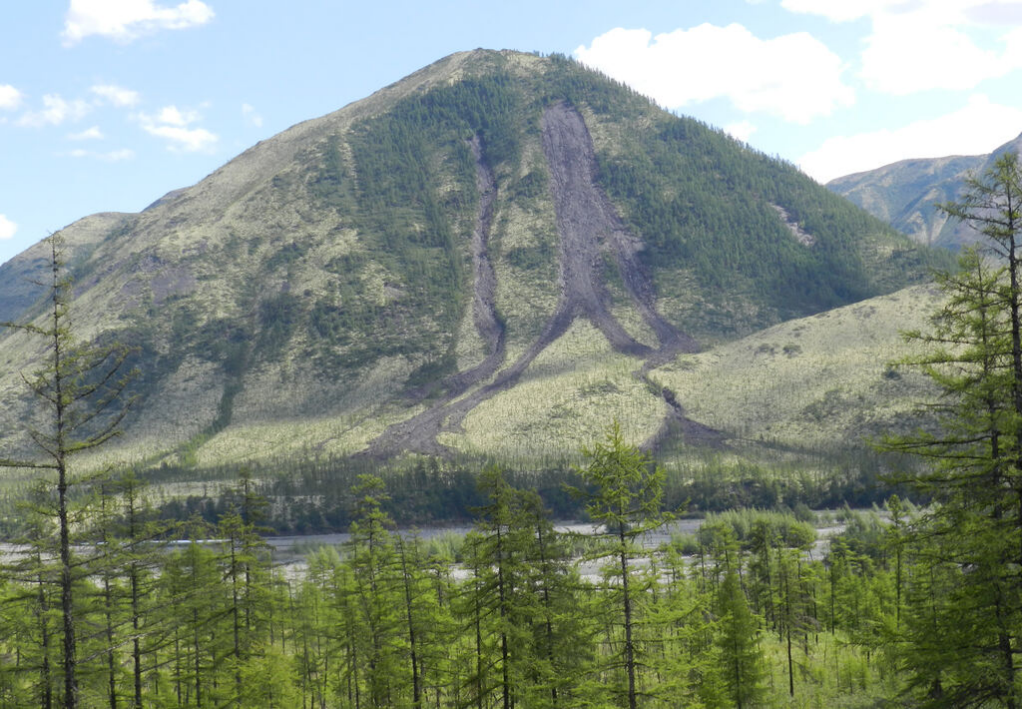
Average aspect of *Larixcajanderi* open forest on steep slopes of Sette-Daban Range. Light green colour implies *Cladoniaalpestris* (L.) Reichenb. dominated forests. Locality 3, 400–800 m a.s.l. Photo of M.S. Ignatov (2015).

Mutual expeditions of the Institute for Biological Problems of Cryolithozone SB RAS and the Main Botanical Garden, Russian Academy of Sciences started in 2000, with the expedition to the southern part of the Region, Yudoma-Maya Plateau and Tarbagannakh Mountain (Ignatov et al. 2001). Subsequent exploration resulted in a number of local moss flora, i.e. of Yana-Adycha Plateau (Isakova 2010), Mus-Khaya Peak surroundings (Ignatova et al. 2011), Orulgan Range (Ignatov et al. 2014), Suntar-Khayata Reserve (Ivanova et al. 2016), Sette-Daban Range (Ignatova et al. 2018), Ust-Nera area (Ivanova et al. 2018) and Ulakhan-Chistai Range (Ignatova et al. 2020). These main collecting localities are shown in Fig. 1. A brief description of the area is as follows. The severe continental climate of the Yana-Indigirka Region makes vegetation quite monotonous; thus, forests here are composed almost exclusively of *Larixcajanderi* Mayr. *Piceaobovata* Ledeb. has just a few populations in the western part of Sette Daban Range (locality 3) and the southern part of the Region (locality 1). River banks have temporary stands of *Populussuaveolens* Fisch. and *Salixarbutifolia* Pall. and wet slopes have a limited occurrence of not very tall trees of *Betulalanata* (Regel) V.N. Vassil. *Pinuspumila* (Pall.) Regel thickets are common, but usually they are composed of shrubs lower than 1.5 m. Despite the low species diversity being found in many of the studied areas (as *Larix* forests are usually dry (Fig. 2), local conditions, make different areas quite distinct in their moss flora, due to different bedrock types and local climates.

1. Ust-Maya District

Collectors: Ignatov M.S., Ivanova E.I.

Collecting year: 2000

Reference: Ignatov et al. 2001

Elevations: 150–1900 m a.s.l.

Coordinates: 60°14'N – 61°08'N; 135°00'E – 138°18'E.

Collection localities were situated in Yudomo-Mayskoe Upland and in foothills of Sette-Daban Range; few collections were made during several short stops along Aldan River.

Bedrocks: dense sandy limestones, dolomites, schists and granites.

Habitats: *Larixcajanderi* forests; flood valley poplar stands; crooked birch forest communities along small creeks; rocks near waterfalls; rock-fields; soil banks, rock outcrops and cliffs; mires.

Number of species: ~250.

Interesting records: *Plagiotheciumberggrenianum* Frisvoll, *Hypnumsaitoi* Ando, *Grimmiatorquata* Drumm., *Platydictyaacuminata* (Lindb. & Arnell) Ignatov and *Didymodonhedysariformis* Otnyukova.

2. Mus-Khaya Mt. surroundings

Collectors: Ignatov M.S., Ignatova E.A., Ivanov O.V., Ivanova E.I.

Collecting year: 2011

Reference: Ignatova et al. 2011

Elevations: 1450–1950 m a.s.l.

Coordinates: 62°31’ – 62°36’N, 140°56’– 141°07’E.

Collection localities: upper course of Kongor Creek and its tributaries.

**Figure 3.**
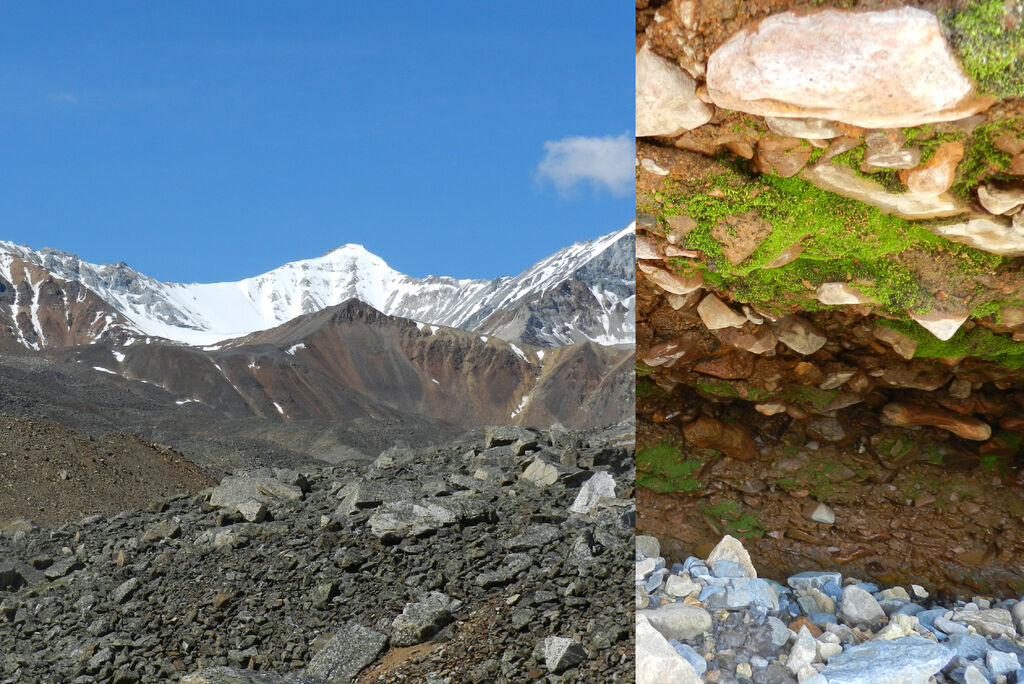
Mus-Khaya Peak (2959 m a.s.l.), lifeless slopes rich in heavy metals. On the right is shown ‘metallothytic’ *Mielichhoferiaasiatica*, one of the few mosses commonly present in similar habitat type. Locality 2, ca. 1600 m a.s.l. Photo of M.S. Ignatov (2011).

Bedrocks are mostly non-carbonate and include schists, aleurolites, sandstones, with granitoid intrusions and acid effusives. Rocks in the area are especially rich in heavy metals (Pb, Sn, As, Zn, Ag, Mn etc.), so rock fields look lifeless, with only scattered patches of mosses (Fig. 3).

**Figure 4.**
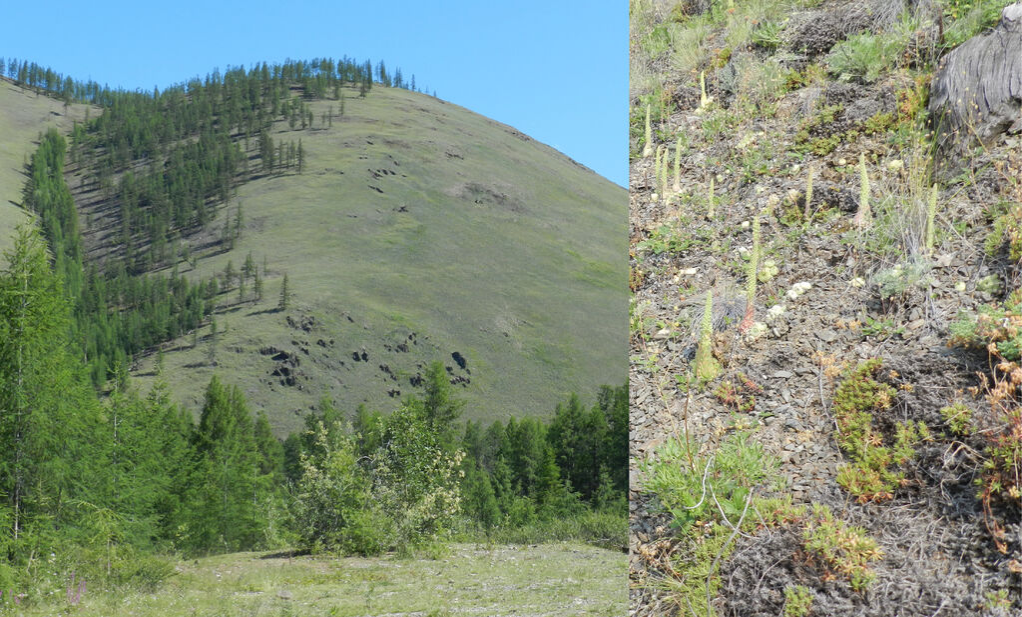
Cryophytic steppes of S-facing slope, with xeric mosses of genera *Pterygoneurum* Jur., *Stegonia* Venturi and *Weissia* Hedw. Locality 2, ca. 800-1000 m a.s.l. Photo of M.S. Ignatov (2011).

Habitats: *Larixcajanderi* forests; mires and bogs (*Sphagnum* communities in wet tundra, at lake shores and in forests; springy fens and hummocks; *Warnstorfia* mires in wet *Carexstans* and *Eriophorumpolystachyon* communities at low banks of small lakes); brook beds; dry lichen tundra; outcrops of various rocks. Steppe communities occur on S-facing steep slopes (Fig. 4).

Number of species: 150.

Interesting records: *Mielichhoferiamielichhoferiana* (Funck) Loeske, *M.elongata* (Hoppe & Hornsch. ex Hook.) Hornsch., *M.asiatica* Tubanova & Ignatova and *Gollaniaturgens* (Müll. Hal.) Ando.

3. Sette-Daban Range

Collectors: Ignatov M.S., Ignatova E.A., Ivanov O.V., Ivanova E.I.

Collecting years: 2015–2017

Reference: Ignatova et al. 2018

Elevations: 350–1550 m a.s.l.

Coordinates: 62°45’ – 63°14’N, 137°03’– 139°01’E.

Collection localities: western slope of Okraina Range; along creeks – right tributaries of Vostochnaya Khandyga River (e.g. Segenyakh Creek); along Kuraanakh, Sakkyryr and Dyby Rivers and their tributaries.

Bedrocks: limestone, mostly metamorphosed to quite solid rocks; Mesozoic sandstones and schists.

Habitats: *Larixcajanderi* forests; thickets of *Pinuspumila*; birch crooked forest and alder shrubs; mountain tundra; rock-fields and limestone cliffs.

Number of species: 294.

Interesting records: *Andreaeobryummacrosporum* Steere & B.M. Murray, *Hydrogoniumamplexifolium* (Mitt.) P.C.Chen, *H.gregarium* (Mitt.) Jan Kučera, *Hymenostyliumxerophilum* Köckinger & Kučera and *Scouleriapulcherrima* Broth.

4. Suntar-Khayata Range

Collectors: Ignatov M.S., Ignatova E.A., Ivanov O.V., Ivanova E.I.

Collecting years: 2015

Reference: Ivanova et al. 2016

Elevations: 800–1490 m a.s.l.

Coordinates: 63°03’ – 63°12’N, 138°48’– 139°27’E.

Collection localities: along creeks – right tributaries of Vostochnaya Khandyga River (Kyurbelyakh and At-Moole Creeks) and Setorym River (Dol and Nekyulyakh Creeks).

Bedrocks: sandstones (with occasional calcareous layers), aleurolites, argillites and schists.

**Figure 5.**
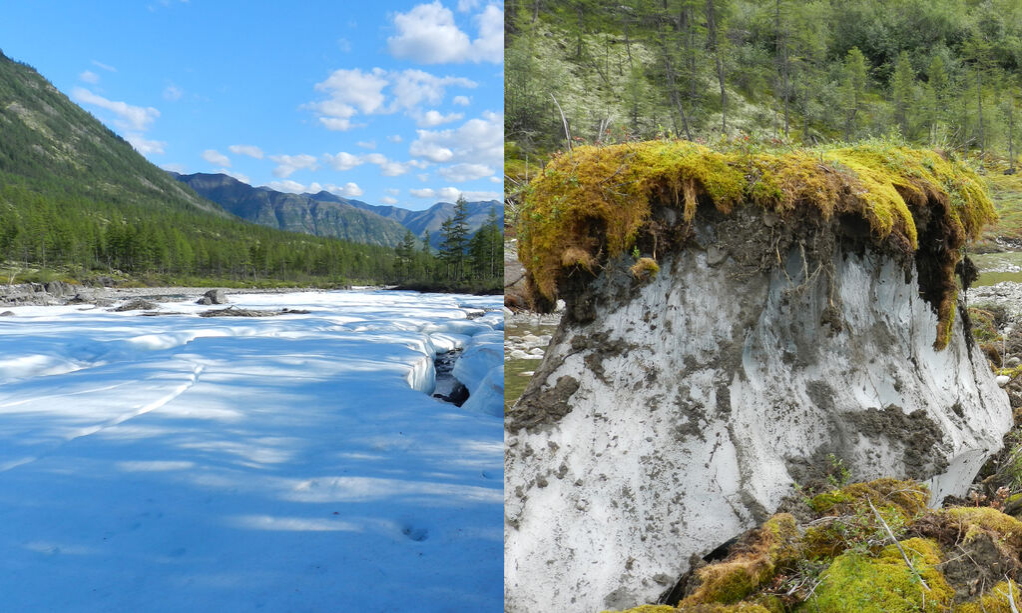
Aufeis glades are common along creeks, lasting to July (left) and leaving only 1(-2) months for moss growth in August (right). Locality 4, ca. 600 m a.s.l. Photo of M.S. Ignatov (2015). Dry rocks are rich in rare xerophytic moss species, for example, *Indusiellathianschanica* Broth. & Müll. Hal., *Didymodonjohansenii* (R.S. Williams) H.A. Crum, *Grimmiatergestina* Tomm. ex Bruch & Schimp., *Pterygoneurumovatum* (Hedw.) Dixon and *Didymodonvinealis* (Brid.) R.H. Zander

Habitats: *Larixcajanderi* forests; mountain tundra; rock-fields and rock outcrops; thickets of *Pinuspumila*; poplar, stone birch and alder communities. Long-lasting or ever-permanent aufeises are common along creeks and rivers (Fig. 5).

Number of species: 208.

Interesting records: *Haplodontiummacrocarpum* (Hook.) J.R. Spence, *Leptopterigynandrumpiliferum* S. He, *Plagiomniumacutum* (Lindb.) T.J. Kop., *Syntrichiapagorum* and Amann *Philonotisfalcata* (Hook.) Mitt.

5. Ust-Nera surroundings

Collectors: Ignatov M.S., Ignatova E.A., Ivanov O.V., Ivanova E.I., Balakirev I.

Collecting years: 2015–2017

Reference: Ivanova et al. 2018

Elevations: 460–1600 m a.s.l.

Coordinates: 64°25’ – 64°42’N, 142°07’– 144°21’E.

Collection localities: surroundings of Ust-Nera Settlement; Ol’chan Pass and Ol’chan gold mine; Nera River valley.

Bedrocks: igneous rocks; aleurolite and argillite interrupted by granite intrusions.

Habitats: *Larixcajanderi* forests; poplar stands; dwarf birch thickets; grass mires and sedge hillocky mires; thickets of *Pinuspumila*, *Alnusfruticosa* and *Betuladivaricata*; rock-fields; steppe communities.

Number of species: 162.

Interesting records: *Pseudotaxiphyllumelegans* (Brid.) Z.Iwats., *Hilpertiavelenovskyi* (Schiffn.) R.H.Zander, *Pterygoneuronkozlovii* Laz.and *Grimmiafuscolutea* Hook.

6. Ulakhan-Chistai Range

Collectors: Ignatov M.S., Ignatova E.A., Ivanov O.V., Ivanova E.I., Balakirev I.

Collecting years: 2018

Reference: Ignatova et al. 2020

Elevations: 575–1300 m a.s.l.

Coordinates: 64°42’ – 65°11’N, 145°32’– 146°45’E.

**Figure 6.**
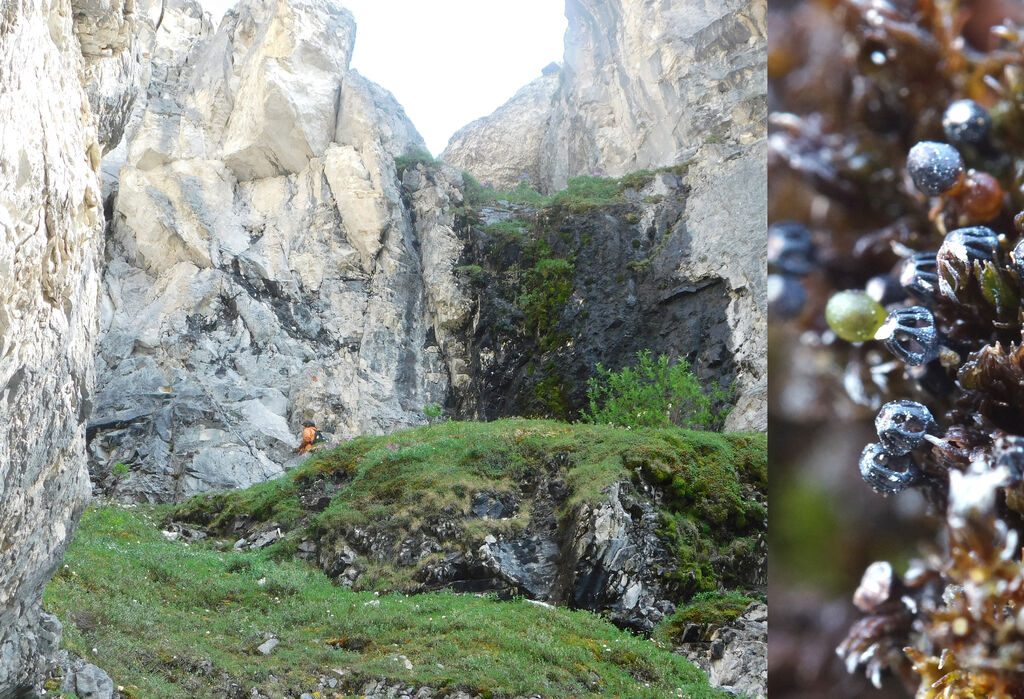
Mramornaya (Marble) Mountain (Ulakhan-Chistai Range), where permanently wet cliff faces are black because of dense cover of relic moss *Andreaeobryummacrosporum* (its close-up is on the right). Locality 6, ca. 900 m a.s.l. Photo of M.S. Ignatov (2018).

Collection localities: Valley of Tirekhtyakh River; Mramornaya Mt. (Fig. 6); Valley of Pravyj Dzhapkychan Creek and surroundings of Kytyp-Kyuel Lake.

Bedrocks: calcareous and acid rocks (marble, siltstone, mudstone, sandstone, slate, granite, rhyolite etc.).

**Figure 7.**
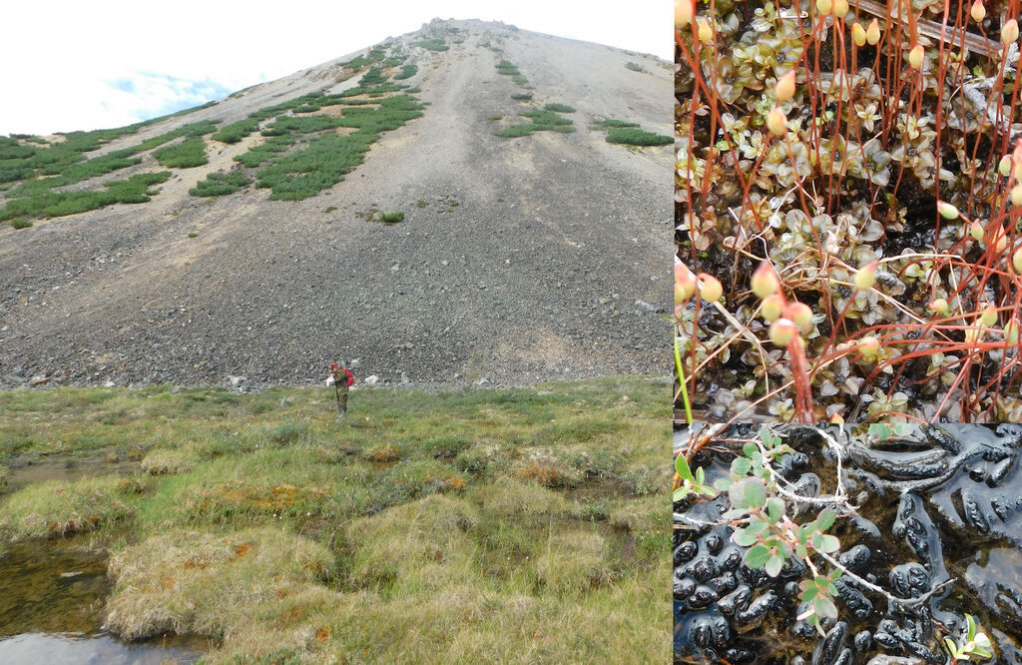
Talus on a hill slope near Kytyp-Kyuel Lake, with scattered *Pinuspumila* procumbent shrubs. Mire at the hill slope lacks any *Sphagna*, being composed of *Cinclidiumstygium* (upper right), *Scorpidiumscorpioides* (lower right) and also *Catoscopium*, *Paludella* and *Meesia*. Locality 6, 1150 m a.s.l. Photo of M.S. Ignatov (2018).

**Figure 8.**
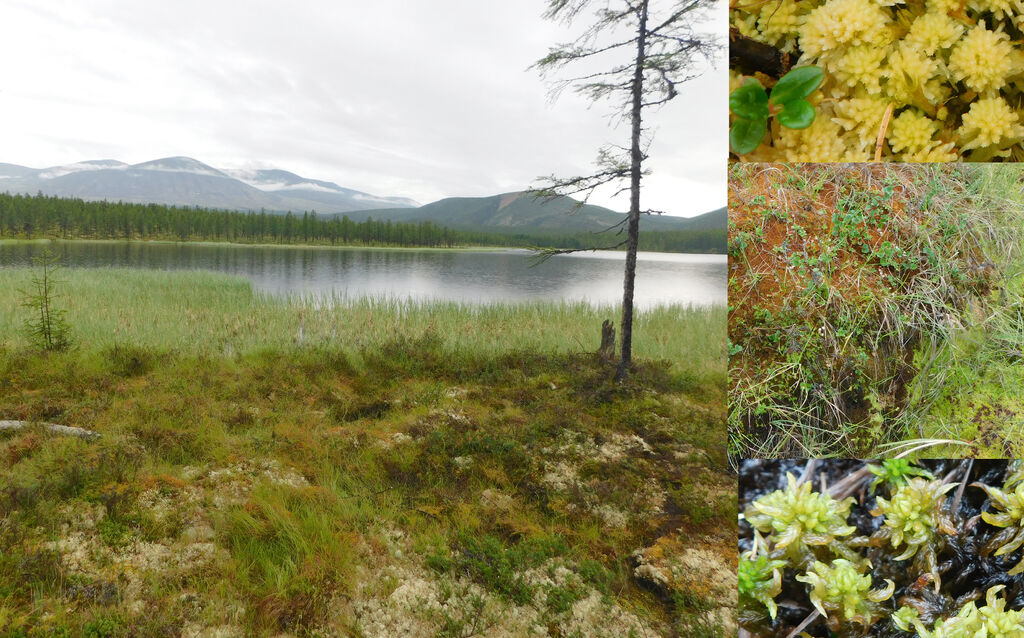
A small Dlinnoe Lake, one of few places were the diverse composition of *Sphagnum* was found. On the right, from top: *Sphagnumaongstroemii* C.Hartm., *S.fuscum* (Schimp.) H.Klinggr. and *S.subsecundum* Nees. Locality 6, ca. 800 m a.s.l. Photo of M.S. Ignatov (2018).

Habitats: *Larixcajanderi* forests; alder and *Pinuspumila* thickets (Fig. 7); various tundra communities in alpine belt and on aufeis glades; mires; lake shores; willow stands in flood valleys; disturbed habitats; places of reindeer and horse grazing; steppe communities; wet marble cliffs, various rock outcrops, rock-fields and dry cliffs. *Sphagnum* bogs are rare (Fig. 8).

Number of species: 325.

Interesting records: *Andreaeobryummacrosporum* Steere & B.M. Murray, *Hilpertiavelenovskyi* (Schiffn.) R.H.Zander, *Didymodongaochenii* B.C. Tan & Y. Jia, *Grimmiatriformis* Carestia & De Not. and*Sphagnummirum* Flatberg & Thingsgaard.

7. Orulgan Range

Collector: Ignatov M.S.

Collecting years: 2011

Reference: Ignatov et al. 2014

Elevations: 450–1750 m a.s.l.

Coordinates: 67°46’ – 68°17’N, 128°06’– 130°50’E.

Collection localities: surroundings of Sakkyryr Settlement; Dyaballakh and Dzhelon Creek valleys; Tumara River Valley; Aenigan-Tolonoo Creek.

Bedrocks: Permian-Triassic aleurolites and schists.

**Figure 9.**
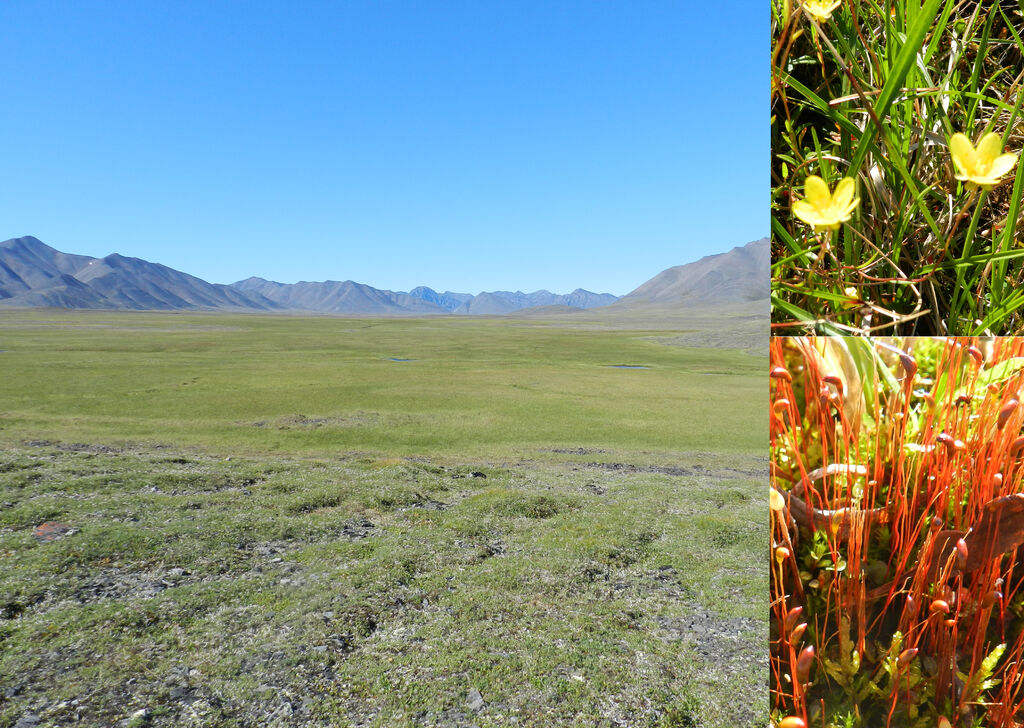
Tumara River upper course: extensive flat community of *Carexstans* Drejer and *Eriophorumangustifolium* Honck., wet due to permafrost melting, providing habitats for *Saxifragahirculus* L. and *Meesiahexasticha*. Locality 7, 1200 m a.s.l. Photo of M.S. Ignatov (2011).

**Figure 10.**
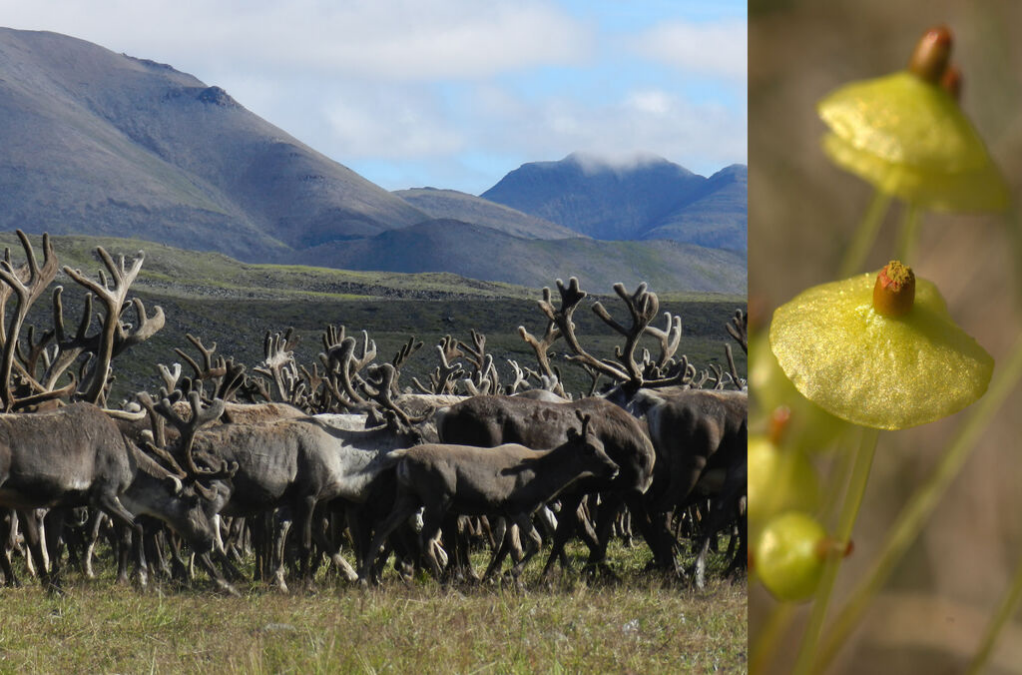
Reindeers in summer pasturing in the mountain tundra in Tumara River upper course. Numerous coprophilous species of Splachnaceae, including *Splachnumluteum* Hedw. (on the right) are relatively common only in areas with extensive reindeer farming. Locality 7, 1200 m a.s.l. Photo of M.S. Ignatov (2011).

Habitats: *Larixcajanderi* forests; tundra communities; rock-fields and rock outcrops; steppe slopes; flat sedge mires with shallow water from melting permafrost (Fig. 9) and reindeer pastures (Fig. 10).

Number of species: 241.

Interesting records: *Indusiellathianschanica* Broth. & Müll.Hal., *Entosthodonpulchellus* (H.Philib.) Brugués, *Bryoerythrophyllumlatinervium* (Holmen) Fedosov & Ignatova, *Didymodonjohansenii* (R.S.Williams) H.A.Crum and *Meesiahexasticha* (Funck) Bruch.

8. Yana – Adycha Plateau

Collector: Isakova V.G.

Collecting years: 2007–2009

Reference: Isakova 2010

Elevations: 130–1726 m a.s.l.

Coordinates: 67°11’ – 68°05’N, 132°47’– 137°06’E.

Collection localities: Verkhoyansk, Batagai, Boronuk, Borulakh, Arylakh, and Ulakhan-Kuel vicinities; Tykakh River Basin; Kikhilyakh Ridge; Mat’-Gora (Ynnakh) mountain.

Bedrocks: Upper Palaeozoic and Mesozoic sedimentary bedrocks, Mesozoic igneous rocks, mainly granites.

Habitats: *Larixcajanderi* forests; *Pinuspumila* thickets; poplar stands; mountain tundra; rock-fields; steppe communities.

Number of species: 173.

Interesting records: *Hilpertiavelenovskyi* (Schiffn.) R.H.Zander, *Pterygoneurumkozlovii* Laz., *Syntrichiacaninervis* Mitt., *Fabroniaciliaris* (Brid.) Brid. and*Coscinodonhartzii* C.E.O. Jensen.

The mountains of Yana-Indigirka Region comprise a great variety of bedrock types. The acid Proterozoic and Permo-Triassic rocks prevail in general, whereas the westernmost and easternmost flanks are formed by pure calcareous ridges. In the west, the Setter-Daban Range faces the Lena River Valley and its main right side tributary, the Aldan River. In the east, the calcareous area, the Mramornaya (Marble) Mountain is a part of the Ulakhan-Chistai Range. Interestingly, these two calcareous regions are the only areas in Eurasia where the ‘living fossil’, the relic monospecific genus, family, order, class and division, *Andreaeobryummacrosporum* Steere & B.M. Murray (Murray 1988, Goffinet et al. 2009), has been discovered. This moss was first collected only in 1974, in Alaska and then described by Steere and Murray 1976 and later found in a few other localities in Alaska, USA and adjacent parts of Canada, in the Yukon, the westernmost Northwest Territories and northern British Columbia (Eckel 2007). In 2015, it was collected in Yakutia, ca. 3000 km from its closest localities in North America (Ignatov et al. 2016) and subsequent intentional search elucidated its distribution in Eurasia (Ignatov et al. 2018).

Other highly isolated populations of mosses, with disjunction more than 1000 km, expand the ranges of many species, for example, *Blindiadelphussubimmersus* (Lindb.) Fedosov & Ignatov, *Didymodonleskeoides* K. Saito, *Haplodontiummacrocarpum* (Hook.) J.R. Spence, *Hydrogoniumamplexifolium* (Mitt.) P.C.Chen, *H.gregarium* (Mitt.) Jan Kučera, *Hymenostyliumxerophilum* Köckinger & Kučera, *Indusiellathianschanica* Broth. & Müll.Hal., *Leptopterigynandrumpiliferum* S. He, *Orthotheciumlapponicum* (Schimp.) C.Hartm., *Philonotisfalcata* (Hook.) Mitt. and *Plagiomniumacutum* (Lindb.) T.J. Kop.

Recently described taxa and still not searchable in GBIF (https://www.gbif.org/species/search, accessed 10 October 2021) comprise an addition to it; those are marked by asterisk (see below).

A large collection from the area allowed us to undertake taxonomic revisions, revealing previously undescribed species: *Barbulajacutica* Ignatova, *Blindiadelphussibiricus Fedosov, *Brachytheciumboreale* Ignatov, *Brachytheciumjacuticum* Ignatov, *Grimmiajacutica* Ignatova, Bedn.-Ochyra, Afonina & J. Muñoz, *Hedwigiaczernyadjevae Ignatova, Ignatov & Fedosov, *Mielichhoferiaasiatica Tubanova & Ignatova, *Orthotheciumbrunnescens Ignatova & Ignatov, *Orthotheciumremotifolium Ignatova & Ignatov, *Orthotheciumretroflexum Ignatova & Ignatov, *Orthotrichumhyperboreum Fedosov & Ignatova, *Schistidiumscabripilum Ignatova & H.H. Blom and *Tomentypnumvittii Hedenäs & Ignatov.

A number of species were also described from adjacent regions and found in Yana-Indigirka Region of Yakutia, some being not rare: *Dicranumbardunovii* Tubanova & Ignatova, *Dicranumschljakovii* Ignatova & Tubanova, *Hedwigiakuzenevae Ignatova & Ignatov, *Orthotheciumsibiricum Ignatov & Ignatova, *Schistidiumrelictum* T.T. McIntosh, H.H. Blom & Ignatova, *Schistidiumtenuinerve* Ignatova & H.H. Blom and *Zygodonsibiricus* Ignatov, Ignatova, Z.Iwats. & B.C.Tan.

Taxonomic revisions, based largely on the rich Yakutian collections also resulted in resurrection of a number of taxa that were incorrectly synonymised earlier: *Scouleriapulcherrima* Broth., *Orthotrichumsibiricum* (Grönvall ex Lindb. & Arnell) Warnst. and *Isopterygiopsiscatagonioides (Broth.) Ignatov & Ignatova. The taxonomic rank of one poorly known taxon was raised to the species level: *Polytrichastrumseptentrionale* (Brid.) E.I.Ivanova, N.E.Bell & Ignatov

This is not totally unexpected, but the fact that undescribed species appeared to be so numerous and some of them are common and widespread in Yakutia, exceeds our expectations. Therefore, it is obvious that further studies in the area will bring numerous very interesting additions to the present collection of data (Ignatov et al. 2021).

## Geographic coverage

### Description

Yakutia, Russia

### Coordinates

60 and 70 Latitude; 123 and 149 Longitude.

## Taxonomic coverage

### Taxa included

**Table:** 

Rank	Scientific Name	
phylum	Bryophyta	

## Traits coverage

### Data coverage of traits

PLEASE FILL IN TRAIT INFORMATION HERE

## Temporal coverage

### Notes

13-06-1910 through 11-07-2019

## Usage licence

### Usage licence

Creative Commons Public Domain Waiver (CC-Zero)

### IP rights notes

This work is licensed under a Creative Commons Attribution (CC-BY) 4.0 License.

## Data resources

### Data package title

MHA Herbarium: Collections of mosses from Yana-Indigirka Region, Yakutia, Russia

### Resource link

https://doi.org/10.15468/je5yds

### Alternative identifiers

10.15468/je5yds,39d71489-2029-4272-95ce-eec6fb8bb5fb, https://depo.msu.ru/ipt/resource?r=yakutia

### Number of data sets

1

### Data set 1.

#### Data set name

MHA Herbarium: Collections of mosses from Yana-Indigirka Region, Yakutia, Russia

#### Data format

Darwin Core

#### Number of columns

44

#### Description

Yana-Indgrirka physiographic region of Yakutia, Russia includes Verkhoyanky Range and some smaller mountain areas; it is one of the largest regions, thus it attracted special attention of bryologists. Several expeditions in 2000-2018 yielded many bryophyte specimens; these data were partly published in a number of papers, but never summarised. This dataset comprehensively represents the diversity of the region. It includes 7,738 records of specimens preserved in the MHA Herbarium.

**Data set 1.** 

Column label	Column description
occurrenceID	An identifier for the Occurrence (as opposed to a particular digital record of the occurrence). A variable that will most closely make the occurrenceID globally unique. A barcode is used for the MHA Herbarium accessions (for example, "MHA9000139").
dcterms:type	The nature or genre of the resource. A constant ("Dataset").
dcterms:modified	The most recent date-time on which the resource was changed. A constant ("17-09-2021").
dcterms:language	A language of the resource. A constant ("en | ru"). English is used throughout and Russian verbatim text is left in "habitat", "county" and "verbatimLocality" fields.
dcterms:licence	A legal document giving official permission to do something with the resource. A constant (http://creativecommons.org/licenses/by/4.0/legalcode).
dcterms:rightsHolder	A person or organisation owning or managing rights over the resource. A constant ("GBS RAN - Glavny Botanichesky Sad Rossijskoj Akademii Nauk").
dcterms:accessRights	Information about who can access the resource or an indication of its security status. A constant ("Use under CC BY 4.0").
institutionID	An identifier for the institution having custody of the object(s) or information referred to in the record. A constant ("http://grbio.org/institution/main-botanical-garden-russian-academy-sciences" for the GBS RAN - Glavny Botanichesky Sad Rossijskoj Akademii Nauk).
collectionID	An identifier for the collection or dataset from which the record was derived. A constant ("urn:lsid:biocol.org:col:15585" for the MHA Herbarium, GBS RAN - Glavny Botanichesky Sad Rossijskoj Akademii Nauk).
datasetID	An identifier for the set of data. May be a global unique identifier or an identifier specific to a collection or institution. A constant ("urn:lsid:biocol.org:col:15585:02").
institutionCode	The name (or acronym) in use by the institution having custody of the object(s) or information referred to in the record. A constant ("GBS RAN - Glavny Botanichesky Sad Rossijskoj Akademii Nauk").
collectionCode	The name, acronym, coden or initialism identifying the collection or dataset from which the record was derived. A constant ("MHA").
datasetName	The name identifying the dataset from which the record was derived. A constant ("MHA Herbarium: Collections of mosses from Yana-Indigirka Region, Yakutia, Russia").
ownerInstitutionCode	The name (or acronym) in use by the institution having ownership of the object(s) or information referred to in the record. A constant ("GBS RAN").
basisOfRecord	The specific nature of the data record - a subtype of the dcterms:type. A constant ("Preserved Specimen").
catalogNumber	An identifier (preferably unique) for the record within the dataset or collection. A variable. A barcode is used for the MHA Herbarium accessions (for example, "MHA9000139").
recordNumber	An identifier given to the Occurrence at the time it was recorded. Often serves as a link between field notes and an Occurrence record, such as a specimen collector's number. A variable (for example, "15-408").
recordedBy	A list (concatenated and separated) of names of people, groups or organisations responsible for recording the original occurrence. A variable. For example, "Ignatov M.S. | Ignatova E.A.".
occurrenceStatus	A statement about the presence or absence of a taxon at a location. A constant ("present").
samplingProtocol	The name of, reference to, or description of the method or protocol used during an Event. A constant ("common practice of herbarium collecting").
eventDate	The date-time or interval during which an Event occurred. For occurrences, this is the date-time when the event was recorded. A variable (for example, "16-07-2015").
habitat	A category or description of the habitat in which the Event occurred. A variable, in Russian (for example, "сырые скальные выходы над галечником").
higherGeography	A list (concatenated and separated) of geographic names less specific than the information captured in the locality term. A variable (for example, "Asia | Russian Federation | Republic of Sakha (Yakutia)").
continent	The name of the continent in which the location occurs. A constant ("Asia").
country	The name of the country or major administrative unit in which the location occurs. A constant ("Russian Federation").
countryCode	The standard code for the country in which the location occurs. A constant ("RU").
stateProvince	The name of the next smaller administrative region than country (state, province, canton, department, region etc.) in which the location occurs. A constant ("Republic of Sakha (Yakutia)").
county	The full, unabbreviated name of the next smaller administrative region than stateProvince (county, shire, department etc.) in which the Location occurs. A variable, in Russian (for example, "Томпонский улус").
verbatimLocality	The original textual description of the place. A variable, in Russian and/or English (for example, "хребет Сетте Дабан; правый берег р. Сегенях (Росомаха) ниже пересечения с Магаданским трактом; Sette-Daban").
minimumElevationInMetres	The lower limit of the range of elevation (altitude, usually above sea level), in metres. De facto, a single figure available on the label is given. A variable (for example, "470").
decimalLatitude	The geographic latitude (in decimal degrees, using the spatial reference system given in geodeticDatum) of the geographic centre of a location. A variable (for example, "63.0417").
decimalLongitude	The geographic longitude (in decimal degrees, using the spatial reference system given in geodeticDatum) of the geographic centre of a location. A variable (for example, "137.95")
geodeticDatum	The ellipsoid, geodetic datum or spatial reference system (SRS) upon which the geographic coordinates given in decimalLatitude and decimalLongitude are based. A constant ("WGS84").
coordinateUncertaintyInMetres	The horizontal distance (in metres) from the given decimalLatitude and decimalLongitude describing the smallest circle containing the whole of the location. A variable (for example, "1000").
coordinatePrecision	A decimal representation of the precision of the coordinates given in the decimalLatitude and decimalLongitude. A constant ("0.0001").
georeferenceRemarks	Notes or comments about the spatial description determination, explaining assumptions made in addition or opposition to the those formalised in the method referred to in georeferenceProtocol. A variable (for example, "by map by Pisarenko").
identifiedBy	A list (concatenated and separated) of names of people, groups or organisations who assigned the Taxon to the subject. A variable (for example, "Ignatova E.A.").
scientificName	The full scientific name, with authorship and date information, if known. A variable (for example, "Abietinella abietina (Hedw.) M.Fleisch.").
kingdom	The full scientific name of the kingdom in which the taxon is classified. A constant ("Plantae").
phylum	The full scientific name of the phylum or division in which the taxon is classified. A constant ("Bryophyta").
taxonRank	The taxonomic rank of the most specific name in the scientificName. A variable (four options: "species", "subspecies", "variety", "genus").
scientificNameAuthorship	The authorship information for the scientificName formatted according to the conventions of the applicable nomenclaturalCode. A variable (for example, "(Hedw.) M.Fleisch.").
nomenclaturalCode	The nomenclatural code (or codes in the case of an ambiregnal name) under which the scientificName is constructed. A constant ("International Code of Nomenclature for algae, fungi and plants").
taxonomicStatus	The status of the use of the scientificName as a label for a taxon. A constant ("accepted").

## Additional information

Ignatov M S, Ignatova E A, Ivanova E A, Isakova V G, Seregin A P (2021). MHA Herbarium: Collections of mosses from Yana-Indigirka Region, Yakutia, Russia. Version 1.1. Tsitsin Main Botanical Garden Russian Academy of Sciences. Occurrence dataset https://doi.org/10.15468/je5yds accessed via GBIF.org on 17-09-2021.
